# COVID-19 and Guillain-Barre Syndrome: a systematic review of case reports

**DOI:** 10.12688/wellcomeopenres.15987.2

**Published:** 2020-09-21

**Authors:** Rodrigo M. Carrillo-Larco, Carlos Altez-Fernandez, Sabrina Ravaglia, Joaquín A. Vizcarra

**Affiliations:** 1 Department of Epidemiology and Biostatistics, School of Public Health, Imperial College London, London, United Kingdom of Great Britain and Northern Ireland, W2 1PG, UK; 2 CRONICAS Centre of Excellence in Chronic Diseases, Universidad Peruana Cayetano Heredia, Lima, Peru; 3 Facultad de Medicina Alberto Hurtado, Universidad Peruana Cayetano Heredia, Lima, Peru; 4 IRCCS C., Mondino Foundation, Pavia, Italy; 5 Department of Neurology, Emory University, Atlanta, USA

**Keywords:** COVID-19, Guillain-Barre Syndrome, neurological complications, pandemic

## Abstract

**Background:** Guillain-Barre Syndrome (GBS) is a neurological autoimmune disease that can lead to respiratory failure and death. Whether COVID-19 patients are at high risk of GBS is unknown. Through a systematic review of case reports, we aimed to summarize the main features of patients with GBS and COVID-19.

**Methods:** Without any restrictions, we searched MEDLINE, Embase, Global Health, Scopus, Web of Science and MedXriv (April 23 rd, 2020). Two reviewers screened and studied titles, abstracts and reports. We extracted information to characterize sociodemographic variables, clinical presentation, laboratory results, treatments and outcomes.

**Results:** Eight reports (n=12 patients) of GBS and COVID-19 were identified; one was a Miller Fisher case. The age ranged between 23 and 77 years, and there were more men (9/102). GBS symptoms started between 5 and 24 days after those of COVID-19. The protein levels in cerebrospinal fluid samples ranged between 40 and 193 mg/dl. None of the cerebrospinal fluid samples tested positive for COVID-19. Six patients debuted with ascendant weakness and three with facial weakness. Five patients had favourable evolution, four remained with relevant symptoms or required critical care and one died; the Miller Fisher case had successful resolution.

**Conclusions:** GBS is emerging as a disease that may appear in COVID-19 patients. Although limited, preliminary evidence appears to suggest that GBS occurs after COVID-19 onset. Practitioners and investigators should have GBS in mind as they look after COVID-19 patients and conduct research on novel aspects of COVID-19. Comparison with GBS patients in the context of another viral outbreak (Zika), revealed similarities and differences that deserves further scrutiny and epidemiological studies.

## Introduction

COVID-19 is a disease for which practitioners and researchers are still learning signs/symptoms, risk factors, co-morbidities and outcomes. Although COVID-19 research is rapidly evolving, novel findings deserve in-depth scrutiny to formulate new hypothesis and make solid conclusions. This is the case of COVID-19 presenting along Guillain-Barre Syndrome (GBS), for which there are a few case reports ^1– 6^.

GBS is a neurological autoimmune disease that can deteriorate hastily, thus requiring high clinical suspicion, early identification and appropriate management. In the past, also in the context of a viral disease outbreak, it has been pinpointed that Zika virus may be a risk factor for GBS ^7– 10^. Whether COVID-19 patients are also at high risk of GBS, is largely unknown. However, the extensive evidence between Zika virus and GBS ^7– 10^, makes it relevant to study and decipher if COVID-19 is also associated with GBS. Consequently, to understand the characteristics of patients with COVID-19 and GBS, and to identify potential patterns, we conducted a systematic review of case reports of COVID-19 and GBS.

## Methods

### Protocol and eligibility criteria

We conducted a systematic review (protocol registration: CRD42020182015) and adhered to the PRISMA guidelines ( *Extended data*: Table S1 ^11^). We searched case reports of COVID-19 and GBS, both as defined by case report. There were no exposures, interventions, comparison groups or specific outcomes, as we aimed to summarize and describe all case reports of COVID-19 and GBS. The patients could have been studied from any healthcare facility.

### Information sources and search

We used six data sources (searched on April 23 ^rd^, 2020): MEDLINE, Embase, Global Health, Scopus and Web of Science (the first three through OVID); we also searched MedRxiv. The search terms are available in *Extended data*: Table S2 ^11^. The search did not include any restrictions. Active surveillance of key neurological journals and academic news helped identify additional sources after the search was conducted.

### Study selection and data collation

Titles, abstracts and full-texts were studied by two reviewers independently (RMC-L and CA-F). Two authors (RMC-L and CA-F) agreed on a data extraction form and piloted it with one report. Extracted information included epidemiological background; disease onset and initial signs/symptoms; laboratory tests and case resolution. The extraction form was not modified during data collection. Data was collected by one reviewer (CA-F) and complemented by others (SR and JV-P).

### Synthesis of results

The extracted information was synthesized qualitatively. Because of the limited number of reports and patients, we did not conduct a quantitative synthesis (e.g., meta-analysis).

### Ethics

This is a systematic review of published case reports. The original reports, nor this work, provided any personal information of the patients. No human subjects were involved in this research. We did not seek authorization by an Ethics Committee.

## Results

### Selection process

**Figure 1.**
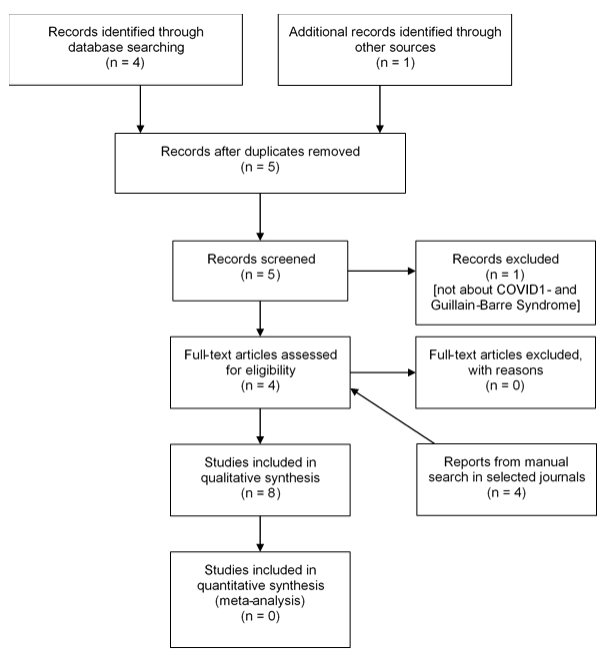
Selection process.

We found 4 reports in OVID and 1 in MedXriv ( Figure 1) ^1– 4, 12^. We did not find any results in Scopus or Web of Science ( Figure 1). In addition, we included 4 reports not yet available in the search results ^5, 6^. Finally, we selected 8 reports (n=12) ^1– 6, 13, 14^. Notably, one patient was a GBS variant: Miller Fisher ^5^.

### Evidence synthesis

The patients were from China (n=1) ^4^, France (n=1) ^14^, Iran (n=1) ^1^, Italy (n=7) ^2, 6, 13^, Spain (n=1) ^5^, and US (n=1) ^3^; the Spanish team reported the Miller Fisher case ^5^.

Overall, the age ranged from 23 to 77 years, and there were more men (9/12) than women ( Table 1).

**Table 1.** Data extracted from the original case reports.

First Author	Virani ^3^	Zhao ^4^	Sedaghat ^1^	Toscano ^2^	Toscano ^2^	Toscano ^2^	Toscano ^2^	Toscano ^2^	Gutierrez-Ortiz ^5^	Padroni ^6^	Camdessanche ^14^	Alberti ^13^
**Country / City**	Pittsburgh / USA	Jingzhou / CHINA	Sari/ IRAN	Pavia / ITALY	Alessandria / ITALY	Brescia / ITALY	Brescia / ITALY	Pavia / ITALY	Madrid/ SPAIN	Romagna/IATLY	Saint-Etienne/ FRANCE	Monza/ITALY
**Sex**	Male	Female	Male	Female	Male	Male	Male	Male	Male	Female	Male	Male
**Age**	54	61	65	77	23	55	76	61	50	70	64	71
**Previous** **comorbidities**	*Not reported*	*Not reported*	Type 2 DM on metformin therapy.	Previous ischemic stroke, diverticulosis	None	Gastric bypass due to obesity	Arterial hypertension, atrial fibrillation on oral anticoagulants	Pericarditis of presumed tubercular origin, 27 years before	Asthma	*Not reported*	*None*	Hypertension, abdominal aortic aneurysm treated with endovascular repair in 2017, and lung cancer treated with surgery only
**Concurrent** **diseases**	Clostridium difficile colitis 2 days before GBS onset	*Not reported*	*Not reported*	Arterial hypertension, atrial fibrillation	*Not reported*	Arterial hypertension, OSAS, metabolic syndrome	Arterial hypertension, atrial fibrillation on oral anticoagulants	Arterial hypertension, thalassaemic trait	*Not reported*	None	None	Severe drug resistant hypertension
**Drugs used** **before GBS** **onset**	Short course amoxicillin + steroids	*Not reported*	HCQ; Lopinavir/ Ritonavir, Azithromycin	Apixaban, bisoprolol, atorvastatin, amlodipine, ramipril	None	*Not reported*	Warfarin; other not reported	Lisinopril	*Not reported*	*Not reported*	*None*	
**COVID-19** **symptoms** **onset**	10 days before GBS onset	7 days after GBS onset	14 days before GBS onset	7 days before GBS onset	10 days before GBS onset	10 days before GBS onset	5 days before GBS onset	7 days before GBS onset	5 days before Miller Fisher variant onset	24 days before GBS onset	11 days before GBS	7 days before GBS without resolution when GBS started
**GBS** **diagnosis**	Clinical diagnosis only	Clinical + CSF analysis + Nerve conduction studies	Clinical + Nerve conduction + Electromyography	Clinical + CSF analysis + Electrophysiological studies	Clinical + CSF analysis + Electrophysiological studies	Clinical + CSF analysis + Electrophysiological studies	Clinical + CSF analysis + Electrophysiological studies	Clinical + CSF analysis + Electrophysiological studies	Miller Fisher variant: Clinical + Serum GD1b-IgG	Clinical + CSF analysis + Electrophysiological studies	Clinical + CSF analysis + Electrophysiological studies	Clinical + CSF analysis + Electrophysiological studies
**Method of** **COVID-19 diagnosis**	RT-PCR	RT-PCR + CT	RT-PCR	RT-PCR	RT-PCR	RT-PCR	RT-PCR	RT-PCR negative in nasopharyngeal swab and BAL; diagnosed by serology	RT PCR	RT-PCR	RT-PCR	RT-PCR
**Autonomic** **symptoms**	Urinary retention	*Not reported*	None	None	None	None	None	None	None	None	None	None
**Blood count**	WBC: 8.6x10 ^3^; HB: 15.4g/dl; PC: 211 x 10 ^3^	Lymphocyte count :0.52 x10 ^9^; Platelet count :113x10 ^9^/L	WBC: 14.6x10 ^3^ (Neutrophils:82.7%, Lymphocytes: 10.4%); HB: 11.6g/dl	WBC: 6.7x10 ^3^ (Lymphocyte: 5.7%)	WBC: 6.32x10 ^3^ (Lymphocyte: 14.7%)	Reported lymphocytopenia (exact value unavailable)	Reported lymphocytopenia (exact value unavailable)	WBC: 10.4x10 ^3^ (Lymphocyte: 13.4%)	Lymphocyte count: 1000cells/UI	WBC: 10.49x10 ^3^	*Not reported*	*Not reported*
**Other lab** **values**	Procalcitonin: 0.15ng/ml	CSF analysis: Cell count = 5x 10 ^6^/L; protein level= 124mg/dl	Glucose: 159; BUN: 19mg/dl; Creatinine: 0.8mg/ dl; ALT: 35UI/L; AST: 47IU/L; Na: 135mmol/L; K: 3.9 mmol/L; ESR: 72mm/hour, CRP: 2+; Urine: negative ketones and glucose	CSF: Day 2: normal protein; no cells; negative PCR for Covid-19 Day 10: protein 101 mg/dl; white-cell count, 4 per mm3; negative PCR assay for COVID-19	CSF: protein level, 123 mg/dl; no cells; negative PCR assay for COVID-19	CSF: protein level, 193 mg/dl; no cells; negative PCR assay for COVID-19	CSF day 5: normal protein level; no cells; negative PCR assay for COVID-19	CSF day 3: protein level, 40 mg/dl; white-cell count, 3 per mm3; negative PCR assay for COVID-19	Serum GD1b-IgG positive. CSF: Opening pressure 11cmH2O, no cells, protein 80mg/dl, glucose 62mg/dl; negative PCR assay for COVID-19	D-dimer, Glucose, Creatinine phosphokinase, hepatic and renal function: All normal CSF: Protein 48mg/dl, cells 1x10 ^6^L. Herpes simplex, varicella zoster, Ebstein Bar virus, CMV, HIV: All negative	CSF: protein level: 166mg/dl, normal cell count Serum: Negative Anti-gangliosides antibodies	CSF: protein level: 54mg/dl; Negative PCR assay for COVID-19, cell count: 9cell/ul
**GBS course**	Ascendant weakness with respiratory failure.	Ascendant weakness with no respiratory failure.	Ascendant weakness and facial bilateral palsy with no respiratory failure.	Flaccid areflexic tetraplegia evolving to facial weakness, upper-limb paraesthesia (36 hr), and respiratory failure (day 6)	Facial diplegia and generalized areflexia evolving to lower limb paraesthesia with ataxia (day 2)	Flaccid tetraparesis and facial weakness evolving to areflexia (day 2) and respiratory failure (day 5)	Flaccid areflexic tetraparesis and ataxia (day 4)	Facial weakness, flaccid areflexic paraplegia (days 2–3), and respiratory failure (day 4)	Miller Fisher variant: right internuclear ophthalmoparesis and right fascicular oculomotor palsy; gait ataxia and loss of tendon reflexes	Ascendant weakness with respiratory failure	Ascendant weakness with respiratory failure	Ascendant weakness with respiratory failure complicated by COVID-19 pneumonia
**Neuropathy** **type**	*Not reported*	Demyelinating	Axonal	Axonal	Axonal	Axonal	Demyelinating	Demyelinating	*Not reported*	Demyelinating	Demyelinating	Demyelinating
**GBS** **Management**	ICU: Mechanical ventilation (4 days) + 400mg/kg IVIG (5 days)	IVIG (dosing not reported)	400mg/kg IVIG (5 days)	400mg/kg IVIG (2 cycles) + temporary mechanical non- invasive ventilation	400mg/kg IVIG	400mg/kg IVIG (2cycles) + mechanical ventilation	400mg/kg IVIG	400mg/kg IVIG + Plasma exchange	400mg/kg IVIG for 5 days.	Not reported	400mg/kg IVIG for 5 days.	400mg/kg IVIG for 5 days.
**COVID-19** **management**	HCQ 400 mg bid for first 2 doses, then 200mg bid for 8 doses	Arbidol, Lopinavir, Ritonavir	HCQ, Lopinavir, Ritonavir, Azithromycin.	Azithromycin (no severe lung disease)	None, no pneumonia	Azithromycin	None, no pneumonia mild respiratory symptoms	None, no pneumonia, symptoms already resolved	*Not reported*	*Not reported*	Acetaminophen, Low molecular weight heparin. lopinavir/ ritonavir 400/100 mg twice a day for ten days	Lopinavir+ Ritonavir and HCQ
**Outcome**	Upper extremities symptoms resolved. Lower extremities weakness persisted; patient was sent to a rehabilitation facility	Symptoms from both GBS and COVID-19 resolved slowly over a 30-day course. Discharged home in day 30	*Not reported*	At week 4: had poor outcomes, including persistence of severe upper- limb weakness, dysphagia, and lower-limb paraplegia	At week 4 had improvements, including decrease in ataxia and mild decrease in facial weakness	At week 4: had poor outcomes, including ICU admission owing to neuromuscular respiratory failure and flaccid tetraplegia	At week 4: had mild improvement but unable to stand 1 month after onset	At week 4: flaccid tetraplegia, dysphagia (enteral nutrition), mechanical invasive ventilation	Complete resolution of Miller Fisher symptoms	At day 8 patient remained in ICU with mechanical invasive ventilation	*Not reported*	The patient died because of progressive respiratory failure.

COVID-19, coronavirus 2019 disease; CSF, cerebrospinal Fluid; EMG, Electromyography, ICU, intensive care unit; IVIG, intravenous immune globulin; RT-PCR, real-time polymerase chain reaction; GBS, Guillain-Barre syndrome; WBC, White blood cell count; PC, platelet count; HB, hemoglobin; BUN, blood urea nitrogen; AST, aspartate transaminase; ALT, alanine transaminase; ESR, erythrocyte sedimentation rate; CRP, c-reactive protein; HCQ, hydroxychloroquine; DM, diabetes mellitus; OSAS, obstructive sleep apnea syndrome; CT, computed tomography; BAL, bronchoalveolar lavage.

In all but one patient, COVID-19 was diagnosed with molecular tests; one patient had the diagnosis made with serological tests ( Table 1) ^2^. In all but one patient, GBS was confirmed with cerebrospinal fluid tests or electromyography ( Table 1). The Miller Fisher case was diagnosed with serum GD1b-IgG ( Table 1) ^5^.

GBS symptoms started between 5–24 days after those of COVID-19 in all but one patient; conversely, in one case, COVID-19 symptoms started 7 days after GBS onset ( Table 1) ^4^. In the Miller Fisher case, COVID-19 symptoms began 5 days before ( Table 1) ^5^.

The earliest cerebrospinal fluid protein levels ranged from 40 mg/dl to 193 mg/dl; protein levels in the Miller Fisher patient was 80 mg/dl ( Table 1) ^5^. All patients whose cerebrospinal fluid was tested for COVID-19, received a negative result ( Table 1).

Among GBS patients, 6 debuted with ascendant weakness and 3 with facial weakness ( Table 1); in addition, 7 patients evolved to respiratory failure between 4 and 6 days after GBS onset ( Table 1).

GBS patients received intravenous immune globulin at 400 mg/kg, and so did the Miller Fisher patient ( Table 1). Regarding COVID-19 treatment, three patients received hydroxychloroquine or other medications, including lopinavir and azithromycin ( Table 1).

Five patients had a favourable outcome with symptoms remission or mild persistent symptoms, four remained with relevant symptoms or required critical care, and one patient died ( Table 1). The Miller Fisher case had successful resolution ( Table 1).

## Discussion

### Main findings

GBS is emerging as a relevant disease that may appear in COVID-19 patients. Male predominance of GBS in COVID-19 patients seems to follow reports about more severe presentation versus its female counterparts. GBS in COVID-19 patients shows heterogeneous presentations both clinical (e.g., ascending or cranial nerve paralysis) and electrophysiological (e.g., axonal or demyelinating). Temporal correlation of GBS seems to occur after COVID-19 onset. Unlike individual case reports, this synthesis of several cases appears to suggest that GBS occurs after COVID-19 onset; nonetheless, this hypothesis deserves further verification with strong epidemiological evidence. Finally, it is too early to determine if the association between GBS and COVID-19 is related to direct viral neurotoxicity, autoimmunity, or both since no validated serological or polymerase chain reaction cerebrospinal fluid tests are commercially available.

### GBS in the context of other viral disease

Although the viral characteristics differ greatly, it is still relevant to make initial comparisons with cases of GBS and Zika virus ( Table 2), where there also appears to be a male predominance and the age profile seems similar ^15, 16^. In both contexts – COVID-19 and Zika – GBS variants with bilateral facial paralysis. On the other hand, cerebrospinal fluid protein levels seem higher in COVID-19 ( Table 2).

**Table 2.** Comparison of GBS in the context of COVID-19 and Zika virus infections.

Characteristics	GBS and Zika virus	GBS and COVID-19
Temporal relationship	Zika symptoms paralleled GBS in 48% of cases ^16^.	In all but one case, COVID-19 symptoms preceded GBS by 5–24 days.
Possible mechanism	Other periinfection mechanisms may be present.	Possible post-inflammatory syndrome.
GBS phenotype	GBS variants with bilateral facial paralysis ^15, 16^.	GBS variants with bilateral facial paralysis.
CSF testing	In 10% of patients RT-PCR was positive in cerebrospinal fluid ^16^.	All cases had a negative RT-PCR in cerebrospinal fluid.
CSF protein levels	Median cerebrospinal fluid protein level: 116mg/dl (IQR=67-171) ^15^.	Cerebrospinal fluid protein level ranged from 40mg/dl to 193mg/dl
Prognosis	Disability at 6 months: mainly facial ^16^.	Not reported.
Other body fluids	Related to long periods of viriuria ^16^.	Not reported.

RT-PCR, real-time polymerase chain reaction; GBS, Guillain-Barre Syndrome; CSF, Cerebrospinal fluid; IQR, Interquartile range.

The experience and management of Zika virus and GBS has provided relevant evidence. It taught us that GBS can be a potential complication during or (shortly) after a viral disease onset. As clinicians receive COVID-19 patients, a neurological examination should not be overlooked at admission and thereafter. Moreover, acknowledging that GBS can be a potential complication of COVID-19 should allow to secure resources (e.g., treatment) to successfully meet the needs of a GBS and COVID-19 patient.

### Research needs

It is still premature to determine a predominance of any of the sociodemographic and clinical features herein summarized. Studies with larger samples and more rigorous design (e.g., retrospective cohorts) are needed to explore this potential association in greater detail to advance the evidence on sociodemographic profiles, clinical presentation and laboratory tests regarding GBS and COVID-19. This way, prognostic factors could be pinpointed so that people at greater risk can be timely managed.

Research comparing GBS associated with COVID-19 and GBS free of COVID-19 ^15^, will also be relevant. We encourage clinicians looking after patients with GBS and COVID-19 to report their experiences; furthermore, we invite them to build networks with colleagues and those whose reports were herein summarized, so that they can conduct more robust studies.

### Limitations

Despite searching six databases, we found few case reports. As it was the case with Zika virus ^8, 17^, more cases may appear later in the pandemic. As the COVID-19 pandemic progresses, clinicians should be aware that GBS and other variants are possible and relevant complications. Our review provides an important first step to better understand the presentation, clinical characteristics and outcomes of COVID-19 and GBS. Epidemiological studies can build on the evidence herein summarised to conduct more robust research.

## Conclusions

GBS is emerging as a relevant neurological disease in COVID-19 patients. Its pathophysiology and both clinical and electrophysiological characteristics remain to be further studied. The GBS onset appears to occur after the COVID-19 presentation by several days. Practitioners and investigators should have GBS in mind as they look after COVID-19 patients and conduct further research on novel aspects of COVID-19.

## Data availability

### Underlying data

All data underlying the results are available as part of the article and no additional source data are required.

### Extended data

Figshare: COVID-19 and Guillain-Barre Syndrome: A systematic review of case reports, https://doi.org/10.6084/m9.figshare.12317486.v2 ^11^.

This project contains the following extended data:

- Table S1: PRISMA checklist.
- Table S2: Search terms.

Data are available under the terms of the Creative Commons Attribution 4.0 International license (CC-BY 4.0).

## References

[1] Sedaghat Z Karimi N: Guillain Barre syndrome associated with COVID-19 infection: A case report. *J Clin Neurosci.* 2020;76:233–235. 10.1016/j.jocn.2020.04.062 32312628 PMC7158817

[2] Toscano G Palmerini F Ravaglia S: Guillain-Barré Syndrome Associated with SARS-CoV-2. *N Engl J Med.* 2020;382(26):2574–2576. 10.1056/NEJMc2009191 32302082 PMC7182017

[3] Virani A Rabold E Hanson T: Guillain-Barré Syndrome associated with SARS-CoV-2 infection. *IDCases.* 2020;e00771. 10.1016/j.idcr.2020.e00771 32313807 PMC7165113

[4] Zhao H Shen D Zhou H: Guillain-Barré syndrome associated with SARS-CoV-2 infection: causality or coincidence? *Lancet Neurol.* 2020;19(5):383–4. 10.1016/S1474-4422(20)30109-5 32246917 PMC7176927

[5] Gutiérrez-Ortiz C Méndez A Rodrigo-Rey S: Miller Fisher Syndrome and polyneuritis cranialis in COVID-19. *Neurology.* 2020;95(5):e601–e605. 10.1212/WNL.0000000000009619 32303650

[6] Padroni M Mastrangelo V Asioli GM: Guillain-Barré syndrome following COVID-19: new infection, old complication? *J Neurol.* 2020;267(7):1877–1879. 10.1007/s00415-020-09849-6 32333166 PMC7180646

[7] Wachira VK Peixoto HM de Oliveira MRF: Systematic review of factors associated with the development of Guillain-Barré syndrome 2007-2017: what has changed? *Trop Med Int Health.* 2019;24(2):132–42. 10.1111/tmi.13181 30444562

[8] Capasso A Ompad DC Vieira DL: Incidence of Guillain-Barré Syndrome (GBS) in Latin America and the Caribbean before and during the 2015-2016 Zika virus epidemic: A systematic review and meta-analysis. *PLoS Negl Trop Dis.* 2019;13(8):e0007622. 10.1371/journal.pntd.0007622 31449532 PMC6730933

[9] Ximenes R Ramsay LC Miranda RN: Health outcomes associated with Zika virus infection in humans: a systematic review of systematic reviews. *BMJ Open.* 2019;9(11):e032275. 10.1136/bmjopen-2019-032275 31685512 PMC6858219

[10] Leonhard SE Mandarakas MR Gondim FAA: Diagnosis and management of Guillain-Barré syndrome in ten steps. *Nat Rev Neurol.* 2019;15(11):671–83. 10.1038/s41582-019-0250-9 31541214 PMC6821638

[11] Rodrigo CL: COVID-19 and Guillain-Barre Syndrome: A systematic review of case reports. *figshare.*Online resource.2020 10.6084/m9.figshare.12317486.v2 PMC7509591 32995555

[12] Bernard-Valnet R Pizzarotti B Anichini A: Two patients with acute meningo-encephalitis concomitant to SARS-CoV-2 infection. *medRxiv.* 2020;20060251 10.1101/2020.04.17.20060251 PMC7267660 32383343

[13] Alberti P Beretta S Piatti M: Guillain-Barre syndrome related to COVID-19 infection. *Neurol Neuroimmunol Neuroinflamm.* 2020;7(4):e741. 10.1212/NXI.0000000000000741 32350026 PMC7217652

[14] Camdessanche JP Morel J Pozzetto B: COVID-19 may induce Guillain-Barre syndrome. *Rev Neurol (Paris).* 2020;176(6):516–518. 10.1016/j.neurol.2020.04.003 32334841 PMC7158797

[15] Dirlikov E Major CG Medina NA: Clinical Features of Guillain-Barré Syndrome With vs Without Zika Virus Infection, Puerto Rico, 2016. *JAMA Neurol.* 2018;75(9):1089–97. 10.1001/jamaneurol.2018.1058 29799940 PMC6143122

[16] Parra B Lizarazo J Jiménez-Arango JA: Guillain-Barré Syndrome Associated with Zika Virus Infection in Colombia. *N Engl J Med.* 2016;375(16):1513–23. 10.1056/NEJMoa1605564 27705091

[17] Mahecha MP Ojeda E Vega DA: Guillain-Barré syndrome in Colombia: where do we stand now? *Immunol Res.* 2017;65(1):72–81. 10.1007/s12026-016-8816-8 27421717

