# Supplementary material for: COVID-19 and Guillain-Barre Syndrome: a systematic review of case reports
Source: Wellcome Open Res. 2020 Sep 21;5:107. Originally published 2020 May 28. [Version 2] doi: 10.12688/wellcomeopenres.15987.2 (PMC7509591; doi:10.12688/wellcomeopenres.15987.2)
Supplement: Peer review history

# Peer review: Reviewer response for version 2

**DOI:** 10.21956/wellcomeopenres.17955.r40538

**Published:** 2020-09-22

**Authors:** Maria Regina Fernandes de Oliveira

**Affiliations:** 1 Centre for Tropical Medicine, University of Brasília, Brasilia, Brazil

I have no new comments. The authors followed the previous recommendations.

Are the rationale for, and objectives of, the Systematic Review clearly stated?

Yes

Is the statistical analysis and its interpretation appropriate?

No

Are sufficient details of the methods and analysis provided to allow replication by others?

Yes

Are the conclusions drawn adequately supported by the results presented in the review?

No

Reviewer Expertise:

Health Tecnology Assessment; Epidemiology; Infectious diseases.

I confirm that I have read this submission and believe that I have an appropriate level of expertise to confirm that it is of an acceptable scientific standard.

# Peer review: Reviewer response for version 1

**DOI:** 10.21956/wellcomeopenres.17533.r40016

**Published:** 2020-09-08

**Authors:** Maria Regina Fernandes de Oliveira

**Affiliations:** 1 Centre for Tropical Medicine, University of Brasília, Brasilia, Brazil

**Paper:** COVID-19 and Guillain-Barre Syndrome: a systematic review of case reports. 

 The research question is truly relevant because of the epidemiological scenario in all the world.   

**Methods:**

 The paper conducted a review of eight reports which describe 12 patients from six countries. The authors summarize some results from 12 patients as median and IQR (ex: median age; median CSF protein levels).  

 Given that the reports came from different populations and different countries, and not represent a homogeneous data set, It’s a methodological mistake to summarize the data in this way. Summarizing the data using these measures could be misleading.

 The data must be presented individually, report by report. The most acceptable is presenting the data range among the reports for the numerical variables or proportions.

Are the rationale for, and objectives of, the Systematic Review clearly stated?

Yes

Is the statistical analysis and its interpretation appropriate?

No

Are sufficient details of the methods and analysis provided to allow replication by others?

Yes

Are the conclusions drawn adequately supported by the results presented in the review?

No

Reviewer Expertise:

Health Tecnology Assessment; Epidemiology; Infectious diseases.

I confirm that I have read this submission and believe that I have an appropriate level of expertise to state that I do not consider it to be of an acceptable scientific standard, for reasons outlined above.

# Response

**Published:** 2020-09-09

**Authors:** Rodrigo M Carrillo-Larco

**Affiliations:** Imperial College London, UK

Dear reviewer,

Thank you very much for taking time and reviewing our work; your input and suggestions are much appreciated.

I appreciate your major comment and understand your concern; however, may I please gently disagree on the following grounds?

1. Your major reservation suggested that our “statistical” approach was not correct, and that we should have not “pooled” the estimates and report means/medias but rather just describe the results (narratively). I think this is a very interesting comment. Nonetheless, we took sort of a “data pooling” approach, in which we summarised, using basic statistics, the main features of the patients. Notably, the individual results were also presented in tables so that the reader could have both, our summaries (means/medians) to have a broad picture of the findings, as well as the results for each patient. We argue that our approach would be similar as if we had accessed the individual-level data of these patients and delivered an individual-level meta-analysis. In that sense, we do not feel our approach was incorrect. 

1. Our approach is not new in the literature, and a quick search of published systematic reviews of case reports in the last few months shows the following:
  1. https://pubmed.ncbi.nlm.nih.gov/32840686/ - this work is an updated version of our research question. And they followed a similar approach reporting, for example: *“…the classical albuminocytological dissociation (cell count < 5/µl with elevated CSF proteins) was detected in 71.2% of the cases (42/59) with a median CSF protein of 100.0 mg/dl…” *As we did, they presented summary measures (median).
  2. https://pubmed.ncbi.nlm.nih.gov/32888662/ - this systematic review of case reports conducted a “ *…exploratory factor analysis of the symptoms was performed.*” This is, arguably, a more complex statistical approach than ours. This could also suggest that one can be more flexible on how to handle the statistical analysis of a systematic review of case reports, with plenty of more options than describing the findings narratively.
  3. https://pubmed.ncbi.nlm.nih.gov/32880011/ - like our work, this review also provided pooled results: “ *…the mean age at presentation was 69.8 years.*”
  4. https://pubmed.ncbi.nlm.nih.gov/32856065/ - this work also provided pooled proportions across all reviewed patients: “ *…with respiratory symptoms being the predominant manifestation (70%)*.”
  5. https://pubmed.ncbi.nlm.nih.gov/32871559/ - similarly, this work also provided pooled means: “ *…The mean age of this population was 25 years (range 2–85 years).*”

I am sure there may be plenty of examples in which the authors decided to conduct a systematic review of case reports and only describe the findings, with no “statistical analysis”. However, we opted for a different approach, in which we gently summarised the findings with simple statistics to provide a broad picture of the overall findings. In addition, the individual findings are provided in tables so that the reader have both: i) a summary of the findings expressed with the aid of basic statistics; and ii) the individual results for each reviewed case (i.e., patient). I believe you raised an interesting point, but I argue that our approach is not incorrect. Moreover, we have provided a few examples suggesting that one can be flexible and conduct some statistical analysis with systematic reviews of cases reports, and this does not invalid the findings. Following these arguments, and if possible, we kindly ask for a reconsideration of your decision.

Again, thank you very much for time in reviewing this work, it is much appreciated. Wish you and your family/friends all the best in these uncertain times.

# Response

**Published:** 2020-09-15

**Authors:** MARIA OLIVEIRA

**Affiliations:** UNIVERSIDADE DE BRASÍLIA, Brazil

Dear authors, 

In your response you argue  "that our approach would be similar as if we had accessed the individual-level data of these patients and delivered an individual-level meta-analysis", but the work didn't perform an individual-level meta-analysis. For such an aproach, please see: Richard D Riley, Paul C Lambert, Ghada Abo-Zaid, "Meta-analysis of individual participant data: rationale,conduct, and reporting". For this rationale, the authors highlight "it is inappropriate to simply analyse individual participant data as if they all came from a single study". On the other hand, there are very few patients from different countries in the reviewed reports, so I suggest not summarize the data as presented. Suppressing medians will not diminish the relevance and quality of the report.

# Peer review: Reviewer response for version 1

**DOI:** 10.21956/wellcomeopenres.17533.r38881

**Published:** 2020-06-25

**Authors:** Hugh J Willison

**Affiliations:** 1 Institute of Infection, Immunity and Inflammation, College of Medical, Veterinary and Life Sciences, University of Glasgow, Glasgow, UK

This review represents a summary of the cases published to date of GBS following COVID-19 infection. The methodology is simply descriptive as the literature in this area is still emerging and case control studies have not been published. It does seem likely from the available reports that typical GBS can follow COVID-19 but that the frequency of this association is uncommon.

Are the rationale for, and objectives of, the Systematic Review clearly stated?

Yes

Is the statistical analysis and its interpretation appropriate?

Not applicable

Are sufficient details of the methods and analysis provided to allow replication by others?

Yes

Are the conclusions drawn adequately supported by the results presented in the review?

Yes

Reviewer Expertise:

GBS and other autoimmune neuropathy.

I confirm that I have read this submission and believe that I have an appropriate level of expertise to confirm that it is of an acceptable scientific standard.
